# Infectious biomarkers upon admission predict in-hospital mortality in COVID-19 patients with and without chronic heart failure

**DOI:** 10.3389/fcimb.2025.1577214

**Published:** 2025-10-24

**Authors:** Rong Wan, Zhaochong Tan, Ying Huang

**Affiliations:** ^1^ Jiangxi Key Laboratory of Molecular Medicine, The Second Affiliated Hospital, Jiangxi Medical College, Nanchang University, Nanchang, Jiangxi, China; ^2^ School of Medicine, Yichun University, Yichun, Jiangxi, China; ^3^ Rehabilitation Department, The Second Affiliated Hospital, Jiangxi Medical College, Nanchang University, Nanchang, Jiangxi, China

**Keywords:** COVID-19, in-hospital mortality, heart failure, inflammation, risk stratification

## Abstract

**Background:**

Coronavirus disease 2019 (COVID-19), caused by severe acute respiratory syndrome coronavirus 2 (SARS-CoV-2), can affect nearly every organ system in the human body and present with diverse clinical manifestations. However, its effects on cardiovascular outcomes remain discrepant.

**Aim:**

The objective of this study was to determine whether blood inflammatory levels on admission were associated with in-hospital mortality risk in patients with congestive heart failure (CHF) and COVID-19.

**Methods:**

We performed a retrospective analysis of 4,711 inpatients with confirmed SARS-CoV-2 infection from the Dryad database. Among these individuals, 541 CHF patients with COVID-19 were compared with hospitalized non-CHF patients (n = 4,170). Admission variables including demographic characteristics, vital signs, preexisting comorbidities, and laboratory indicators were obtained as potential confounders for in-hospital mortality risk.

**Results:**

Univariate analysis with Kaplan–Meier curves suggested that higher inflammatory levels on admission—including white blood cell (WBC) count, interleukin-6 (IL-6), ferritin, procalcitonin, and C-reactive protein—were associated with a significantly higher risk of in-hospital mortality compared with lower levels. Consistently, multivariate Cox regression analysis showed that apart from ferritin [0.8 (0.7, 0.9), <0.001], the WBC [1.3 (1.1, 1.5), 0.013], IL-6 [1.4 (1.2, 1.7), <0.001], procalcitonin [1.2 (1.0, 1.3), 0.031] and C-reactive protein [1.2 (1.1, 1.4), 0.002] were independently associated with increased risk of in-hospital mortality in non-CHF patients in the adjusted II model. However, these independent relationships were not observed in CHF patients.

**Conclusion:**

Elevated systemic inflammatory levels on admission were significantly associated with increased in-hospital mortality risk in non-CHF patients with COVID-19 but not in CHF patients. These findings may provide a clinical basis for risk stratification in CHF patients. Further studies are needed to support these results.

## Introduction

Coronavirus disease 2019 (COVID-19), caused by severe acute respiratory syndrome coronavirus 2 (SARS-CoV-2) (Stalder and Thiel, 2021; Jackson et al., 2022), has undergone continuous mutation, leading to varied clinical manifestations with high morbidity and mortality, particularly among elderly patients with chronic underlying conditions such as cardiovascular and respiratory diseases (Rey et al., 2020). SARS-CoV-2 can invade myocardial cells via the angiotensin-converting enzyme 2 (ACE2) receptor, resulting in acute cardiovascular events such as myocarditis and acute myocardial infarction (MI), which may subsequently lead to congestive heart failure (CHF), arrhythmia, and even cardiac arrest (Guzik et al., 2020; Liu et al., 2020). In addition, the virus can induce a severe inflammatory cytokine storm or immune response, causing further damage to myocardial tissue and coronary arteries (Guzik et al., 2020; Liu et al., 2020). Previous studies have demonstrated that patients with confirmed COVID-19 and heart failure (HF) face an increased risk of morbidity and mortality (Panhwar et al., 2019; Isath et al., 2023). A recent high-quality study also reported that COVID-19 infection in patients with acute HF is significantly associated with increased in-hospital mortality (Hashem et al., 2023). Inflammatory markers such as neutrophil-to-lymphocyte ratio (NLR) and monocyte-to-lymphocyte ratio (MLR) have been correlated with mortality risk in non-critically ill patients with COVID-19 (Guzik et al., 2020; Liu et al., 2020). However, other inflammatory markers—including interleukin-6 (IL-6), ferritin, C-reactive protein, and procalcitonin—have not been fully explored in these patients. Moreover, little evidence exists on the association between systemic inflammatory levels on admission and in-hospital mortality risk in CHF patients with confirmed COVID-19 (Huang et al., 2022; Xie et al., 2022).

Therefore, we analyzed a large sample of inpatients with confirmed SARS-CoV-2 infection (n = 4,711) from the Dryad database to assess whether blood levels of inflammatory biomarkers—white blood cell (WBC) count, IL-6, ferritin, C-reactive protein, and procalcitonin—on admission serve as independent predictors of in-hospital mortality risk among CHF and non-CHF patients.

## Methods

### Study population

Our study data were obtained from the Dryad database (https://doi.org/10.5061/dryad.7d7wm37sz) where a retrospective study including a total of 4,711 patients with confirmed SARS-CoV-2 infection who were admitted to four hospitals within the Montefiore Health System between March 1 and April 16, 2020 (Altschul, 2021; Eskandar et al., 2021). Clinical information, including demographic characteristics, preexisting comorbidities, admission vital signs, admission laboratory indicators, medications, and in-hospital mortality, was confirmed by the healthcare surveillance software package (Clinical Looking Glass; Streamline Health, Atlanta, GA) and by review of the primary medical records (Altschul et al., 2020).

Inclusion criteria: all patients with confirmed SARS-CoV-2 infection tested by real-time reverse transcriptase PCR assay were included.

Exclusion criteria: 1) patients who died before admission or were not admitted were excluded because they did not have a complete set of laboratory indicators; 2) only the last report was considered for analysis if patients had multiple admissions. For the purpose of this study, 4,711 patients were finally enrolled in the analysis. According to the regulations of the Dryad database, we registered and then obtained the dataset for free for further analysis without additional ethical consent.

### In-hospital mortality and admission variables

To achieve the purpose of this study, in-hospital mortality was considered the primary outcome. Mortality data were collected by querying in-hospital deaths and deaths recorded in the National Death Registry. Only laboratory indicators obtained at admission were included for analysis. Comorbidities were identified by the International Classification of Diseases, 10th Revision (ICD-10), and the comorbidities chosen were those used in the Charlson Comorbidity Index (Sasson et al., 2017; Altschul et al., 2020; Eskandar et al., 2021). Each patient’s medical record was reviewed for diagnoses occurring within 5 years.

### Statistical analysis

To evaluate the relevance of inflammatory indicators at admission with in-hospital mortality risk among CHF and non-CHF patients with COVID-19, all patients were divided into two subgroups (with CHF and without CHF). Mean ± standard deviation (SD) was used for the statistical description of continuous variables with a normal distribution and was compared by an independent t-test. Median (25–75 percentiles) was used for continuous variables with a non-normal distribution and was compared using the Mann–Whitney U test. The N (%) was used for categorical variables and was compared using Pearson’s chi-square test. Firstly, univariate Kaplan–Meier curve analysis was performed to evaluate in-hospital mortality risk in these patients. Then, to confirm independent associations of inflammatory indicators (WBC, IL-6, ferritin, C-reactive protein, and procalcitonin) with in-hospital mortality risk, multivariate logistic regression analysis was performed using odds ratios (ORs) with 95% confidence intervals (CIs). To further confirm independent predictors of in-hospital mortality risk, multivariate Cox regression analysis was conducted using these admission inflammatory indicators as exposure variables, associated with the primary outcome by hazard ratios (HRs) with 95% CIs. The confounding factors were as follows: 1) Adjusted I included age, Black, White, Asian, and Latino; 2) Adjusted II included age, Black, White, Asian, Latino, temperature, mean arterial pressure, and oxygen saturation.

We finally used sensitivity analysis to evaluate the predictive value of inflammatory indicators at admission for in-hospital mortality risk. The number of days from admission to in-hospital death was used as the time-to-event data. For all statistical analyses in this study, a p-value of less than 0.05 was considered statistically significant. All statistical analyses were performed using Empower (version 4.1) or SPSS (version 24.0).

## Results

### Admission characteristics (baseline)

As shown in **Table 1**, among the 4,711 patients with confirmed COVID-19 infection who were recorded in the Montefiore Health System, 541 (11.5%) individuals were diagnosed with CHF. These CHF patients were older and had a higher prevalence of preexisting comorbidities, including MI, peripheral vascular disease (PVD), cerebrovascular disease (CVD), dementia, chronic obstructive pulmonary disease (COPD), complicated diabetes mellitus, uncomplicated diabetes mellitus, renal disease, and abnormal liver function [aspartate aminotransferase (AST) and alanine aminotransferase (ALT)], compared with those without CHF (n = 4,170). Interestingly, CHF patients did not have worse outcomes in admission vital signs (oxygen saturation, temperature, and mean arterial pressure), inflammatory indicators at admission [ferritin, C-reactive protein, and procalcitonin, but not WBC, lymphocytes, or IL-6], or in-hospital mortality compared with non-CHF patients (all p > 0.05). In addition, there was no significant difference in ethnic group distribution between these two subgroups (p > 0.05).

**Table 1:** Admission characteristics.

Variables	Non-CHF (N=4170)	CHF (N=541)	P value
Age (Years)	63.19 ± 16.74	64.79 ± 16.38	0.049
Ethnic groups			
Black, n (%)	1528 (36.64%)	215 (39.74%)	0.160
White, n (%)	402 (9.64%)	64 (11.83%)	0.108
Asian, n (%)	109 (2.61%)	12 (2.22%)	0.584
Latino, n (%)	1565 (37.53%)	188 (34.75%)	0.208
Vital signs			
Oxygen saturation (%)	89.72 ± 18.81	88.63 ± 20.15	0.140
Temperature (°C)	35.81 ± 7.70	35.80 ± 8.04	0.103
Mean arterial pressure (mmHg)	81.84 ± 24.45	81.02 ± 24.83	0.409
Preexisting comorbidities			
MI, n (%)	74 (1.77%)	127 (23.48%)	<0.001
PVD, n (%)	605 (14.51%)	208 (38.45%)	<0.001
CVD, n (%)	329 (7.89%)	177 (32.72%)	<0.001
Dementia, n (%)	250 (6.00%)	122 (22.55%)	<0.001
COPD, n (%)	173 (4.15%)	92 (17.01%)	<0.001
Diabetes mellitus complicated, n (%)	328 (7.87%)	167 (30.87%)	<0.001
Diabetes mellitus simple, n (%)	422 (10.12%)	264 (48.80%)	<0.001
Renal disease, n (%)	480 (11.51%)	353 (65.25%)	<0.001
Laboratory measurement			
D-Dimer (mg/liter)	3.24 ± 5.28	3.10 ± 5.21	0.185
Platelets (k per mm3)	227.89 ± 116.40	214.88 ± 104.30	0.019
Creatinine (µmol/liter)	1.93 ± 2.63	2.09 ± 2.73	0.016
BUN (mg/dL)	26.98 ± 31.24	28.89 ± 31.03	0.029
International normalized ratio	1.10 ± 1.01	1.12 ± 0.89	0.164
Glucose (mmol/liter)	128.59 ± 132.92	121.79 ± 115.16	0.723
AST (U/liter)	62.45 ± 205.81	69.01 ± 194.14	0.002
ALT (U/liter)	42.28 ± 110.06	47.20 ± 100.34	0.081
WBC (per mm3)	8.52 ± 7.05	8.11 ± 9.37	0.013
Lymphocytes (per mm3)	1.31 ± 4.61	1.33 ± 6.49	0.009
IL-6 (pg/ml)	136.66 ± 2378.82	35.43 ± 104.10	0.050
Ferritin (µg/liter)	1049.79 ± 3204.77	1109.88 ± 2081.58	0.553
C-reactive protein (mg/liter)	10.32 ± 11.22	10.30 ± 11.28	0.876
Procalcitonin (ng/ml)	1.54 ± 6.17	1.93 ± 6.94	0.459
In-hospital mortality (%)	998 (23.93%)	150 (27.73%)	0.053
Hospitalization time (Length of stay, days)	7.12 ± 6.99	7.47 ± 7.33	0.662

CHF, congestive heart failure; MI, myocardial infarction; PVD, peripheral vascular disease; CVD, cerebrovascular disease; COPD, chronic obstructive pulmonary disease; BUN, urea nitrogen; AST, aspartate aminotransferase; ALT, alanine aminotransferase; WBC, white blood cell count; IL-6, interleukin-6.

### Multivariate logistic regression analysis

We first applied multivariate logistic regression analysis to evaluate the potential associations between inflammatory biomarkers at admission and in-hospital mortality risk after adjusting for sociodemographics and vital signs (**Table 2**). The adjusted I model showed that, in addition to WBC and IL-6, the inflammatory biomarkers ferritin, procalcitonin, and C-reactive protein were significantly and independently associated with a higher rate of in-hospital mortality after adjusting for age and ethnic group in both CHF and non-CHF patients. Consistently, after further adjusting for age, ethnic group, and vital signs, the adjusted II model suggested that, in addition to WBC and IL-6, ferritin, procalcitonin, and C-reactive protein remained associated with a higher rate of in-hospital mortality in the two subgroups.

**Table 2:** Multivariate logistic regression analysis for the association of blood inflammatory markers with in-hospital mortality risk.

Exposure	Non-CHF OR (95% CI), P value	CHF OR (95% CI), P value	Total OR (95% CI), P value
Adjusted I			
WBC (per mm3)	1.0 (1.0, 1.0), <0.001	1.0 (1.0, 1.0), 0.778	1.0 (1.0, 1.0), <0.001
IL-6 (pg/ml)	1.0 (1.0, 1.0), 0.077	1.0 (1.0, 1.0), <0.001	1.0 (1.0, 1.0), 0.072
Ferritin (µg/liter)	1.0 (1.0, 1.0), <0.001	1.0 (1.0, 1.0), 0.005	1.0 (1.0, 1.0), <0.001
Procalcitonin (ng/ml)	1.1 (1.0, 1.1), <0.001	1.1 (1.0, 1.1), <0.001	1.1 (1.0, 1.1), <0.001
C-reactive protein (mg/liter)	1.0 (1.0, 1.0), <0.001	1.1 (1.0, 1.1), <0.001	1.0 (1.0, 1.0), <0.001
Adjusted II			
WBC (per mm3)	1.0 (1.0, 1.0), 0.028	1.0 (0.9, 1.0), 0.657	1.0 (1.0, 1.0), 0.040
IL-6 (pg/ml)	1.0 (1.0, 1.0), 0.635	1.0 (1.0, 1.0), <0.001	1.0 (1.0, 1.0), 0.629
Ferritin (µg/liter)	1.0 (1.0, 1.0), 0.003	1.0 (1.0, 1.0), 0.055	1.0 (1.0, 1.0), 0.001
Procalcitonin (ng/ml)	1.1 (1.0, 1.1), <0.001	1.1 (1.0, 1.1), <0.001	1.1 (1.0, 1.1), <0.001
C-reactive protein (mg/liter)	1.0 (1.0, 1.0), <0.001	1.0 (1.0, 1.1), <0.001	1.0 (1.0, 1.0), <0.001

Adjusted I: Age, Black, White, Asian and Latino.

Adjusted II: Age, Black, White, Asian, Latino, temperature, mean arterial pressure and oxygen saturation.

CHF, congestive heart failure; WBC, white blood cell count; IL-6, interleukin-6.

Furthermore, we divided these five biomarkers into binary variables to estimate in-hospital mortality risk (**Table 3**). We observed that elevated inflammatory levels—including WBC (<4,800 or >10,800 per mm³), IL-6 (>150 pg/ml), procalcitonin (>0.1 ng/ml), and C-reactive protein (>10 mg/l)—were significantly associated with a higher rate of in-hospital mortality after controlling for age, Black, White, Asian, Latino, temperature, mean arterial pressure, and oxygen saturation in all patients (CHF and non-CHF), as shown in the adjusted II model.

**Table 3:** Multivariate logistic regression analysis for the association of blood inflammatory markers with in-hospital mortality risk.

Exposure	Non-CHF OR (95% CI), P value	CHF OR (95% CI), P value	Total OR (95% CI), P value
Adjusted I			
WBC <4800 or > 10,800 per mm3	1.6 (1.2, 1.9), <0.001	0.9 (0.5, 1.4), 0.635	1.4 (1.2, 1.7), <0.001
IL-6 > 150 pg/ml	3.5 (2.6, 4.5), <0.001	6.7 (3.0, 14.8), <0.001	3.7 (2.9, 4.8), <0.001
Ferritin > 300 µg/liter	1.2 (1.1, 1.4), 0.006	1.4 (1.0, 2.2), 0.079	1.3 (1.1, 1.5), 0.001
Procalcitonin > 0.1 ng/ml	2.5 (2.1, 2.9), <0.001	3.4 (2.2, 5.1), <0.001	2.6 (2.2, 2.9), <0.001
C-reactive protein > 10 mg/liter	2.3 (2.0, 2.7), <0.001	2.8 (1.8, 4.2), <0.001	2.4 (2.1, 2.7), <0.001
Adjusted II			
WBC <4800 or > 10,800 per mm3	1.4 (1.1, 1.8), 0.003	0.7 (0.4, 1.2), 0.147	1.3 (1.0, 1.6), 0.021
IL-6 > 150 pg/ml	2.8 (2.1, 3.7), <0.001	4.9 (2.0, 12.2), <0.001	2.9 (2.2, 3.9), <0.001
Ferritin > 300 µg/liter	1.0 (0.9, 1.2), 0.689	1.4 (0.9, 2.2), 0.172	1.1 (0.9, 1.2), 0.442
Procalcitonin > 0.1 ng/ml	2.1 (1.8, 2.5), <0.001	3.0 (1.8, 4.7), <0.001	2.2 (1.9, 2.6), <0.001
C-reactive protein > 10 mg/liter	1.9 (1.6, 2.2), <0.001	2.3 (1.4, 3.6), <0.001	1.9 (1.6, 2.2), <0.001

Adjusted I: Age, Black, White, Asian and Latino.

Adjusted II: Age, Black, White, Asian, Latino, temperature, mean arterial pressure and oxygen saturation.

CHF, congestive heart failure; WBC, white blood cell count; IL-6, interleukin-6.

### Multivariate Cox regression analysis

**Figure 1:**
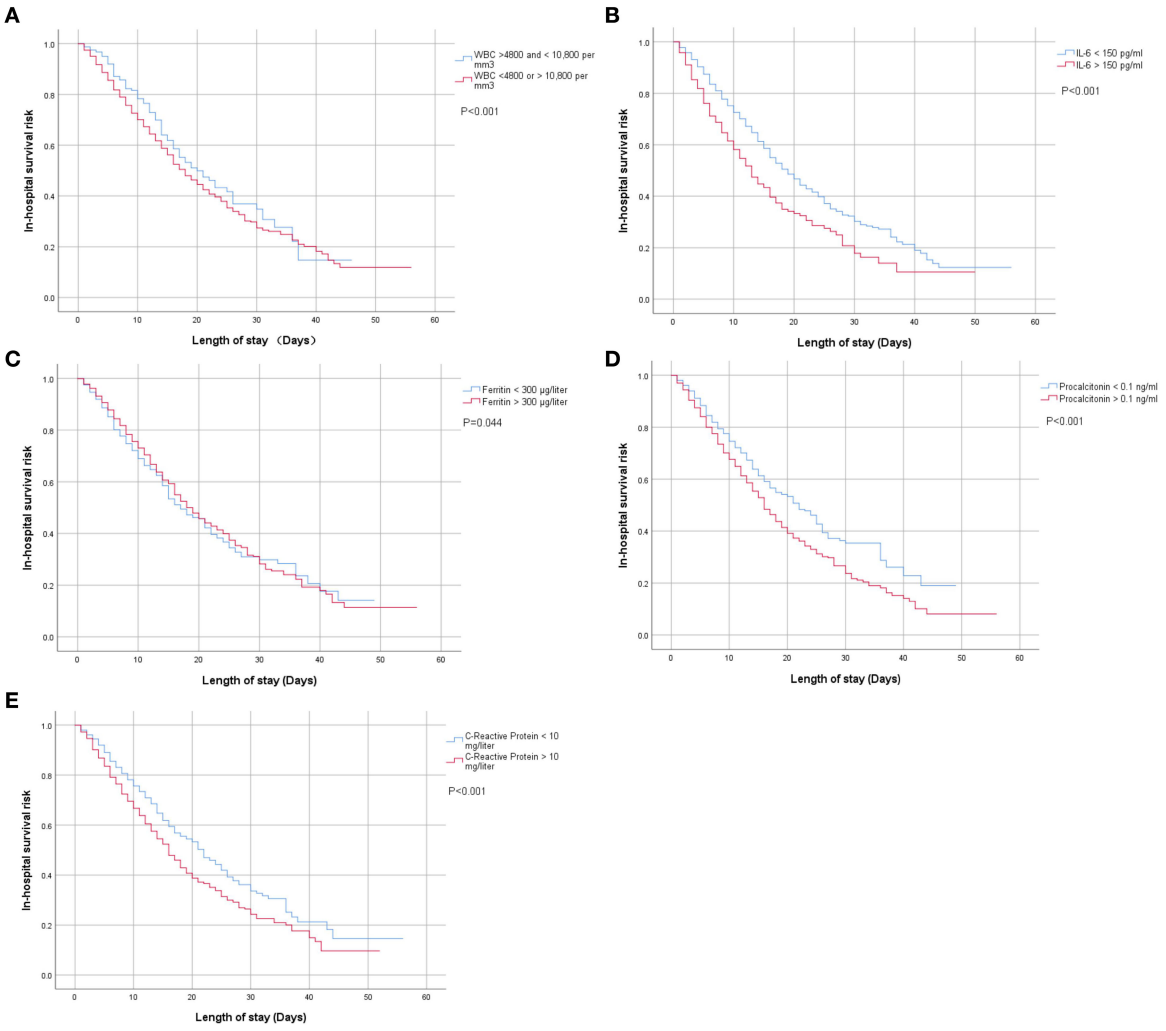
**(A–E)** Univariate Kaplan–Meier analysis of the associations between blood inflammatory biomarkers at admission and in-hospital mortality risk among patients with confirmed COVID-19 infection.

The univariate analysis of Kaplan–Meier curves suggested that higher inflammatory levels—including WBC (<4,800 or >10,800 per mm3), IL-6 (>150 pg/ml), ferritin (>300 µg/l), procalcitonin (>0.1 ng/ml), and C-reactive protein (>10 mg/l)—were associated with a significantly higher risk of in-hospital mortality compared with lower levels [WBC (>4800 and <10,800 per mm3), IL-6 (<150 pg/ml), ferritin (< 300 µg/liter), procalcitonin (<0.1 ng/ml) and C-reactive protein (<10 mg/l)], as shown in **Figures 1A–E**.

Findings were consistent with the above results. Multivariate Cox regression analysis in the adjusted II model demonstrated that, in addition to ferritin [0.8 (0.7, 0.9), <0.001], WBC [1.3 (1.1, 1.5), 0.013], IL-6 [1.4 (1.2, 1.7), <0.001], procalcitonin [1.2 (1.0, 1.3), 0.031], and C-reactive protein [1.2 (1.1, 1.4), 0.002] contributed to a higher risk of in-hospital mortality in non-CHF patients but not in CHF patients (**Table 4**).

**Table 4:** Multivariate Cox regression analysis for the association of blood inflammatory markers with in-hospital mortality risk.

Exposure	Non-CHF HR (95% CI), P value	CHF HR (95% CI), P value	Total HR (95% CI), P value
Adjusted I			
WBC <4800 or > 10,800 per mm3	1.4 (1.1, 1.6), 0.002	0.9 (0.6, 1.3), 0.527	1.3 (1.1, 1.5), 0.008
IL-6 > 150 pg/ml	1.6 (1.3, 2.0), <0.001	1.4 (0.9, 2.2), 0.177	1.6 (1.3, 1.9), <0.001
Ferritin > 300 µg/liter	0.9 (0.8, 1.0), 0.062	0.9 (0.6, 1.3), 0.536	0.9 (0.8, 1.0), 0.071
Procalcitonin > 0.1 ng/ml	1.3 (1.2, 1.5), <0.001	1.2 (0.9, 1.7, 0.221	1.3 (1.2, 1.5), <0.001
C-reactive protein> 10 mg/liter	1.4 (1.2, 1.6), <0.001	1.5 (1.1, 2.1), 0.020	1.4 (1.3, 1.6), <0.001
Adjusted II			
WBC <4800 or > 10,800 per mm3	1.3 (1.1, 1.5), 0.013	0.8 (0.5, 1.2), 0.238	1.2 (1.0, 1.4), 0.053
IL-6 > 150 pg/ml	1.4 (1.2, 1.7), <0.001	1.1 (0.7, 1.7), 0.741	1.4 (1.1, 1.6), <0.001
Ferritin > 300 µg/liter	0.8 (0.7, 0.9), <0.001	0.8 (0.6, 1.2), 0.255	0.8 (0.7, 0.9), <0.001
Procalcitonin > 0.1 ng/ml	1.2 (1.0, 1.3), 0.031	1.1 (0.8, 1.5), 0.618	1.2 (1.0, 1.3), 0.022
C-reactive protein > 10 mg/liter	1.2 (1.1, 1.4), 0.002	1.2 (0.8, 1.7), 0.292	1.2 (1.1, 1.4), 0.001

Adjusted I: Age, Black, White, Asian and Latino.

Adjusted II: Age, Black, White, Asian, Latino, temperature, mean arterial pressure and oxygen saturation.

CHF, congestive heart failure; WBC, white blood cell count; IL-6, interleukin-6.

### Sensitivity analysis

Further sensitivity analysis suggested that WBC (<4800 or >10,800 per mm3), IL-6 (>150 pg/ml), ferritin (>300 µg/liter), procalcitonin (>0.1 ng/ml) and C-reactive protein (>10 mg/liter) were still associated with in-hospital mortality risk in non-CHF patients but not in CHF patients by adjusted for age, Black, White, Asian, Latino, temperature, mean arterial pressure, oxygen saturation, MI, PVD, CVD, dementia, COPD, diabetes mellitus complicated and renal disease (**Table 5**).

**Table:** Sensitivity analysis.

Exposure	Non-CHF HR (95% CI), P value	CHF HR (95% CI), P value	Total HR (95% CI), P value
Adjusted II			
WBC <4800 or > 10,800 per mm3	1.3 (1.1, 1.6) 0.011	0.8 (0.5, 1.2) 0.201	1.2 (1.0, 1.4) 0.055
IL-6 > 150 pg/ml	1.4 (1.2, 1.7) <0.001	1.1 (0.7, 1.8) 0.776	1.4 (1.1, 1.6) <0.001
Ferritin > 300 µg/liter	0.8 (0.7, 0.9) <0.001	0.8 (0.6, 1.2) 0.345	0.8 (0.7, 0.9) <0.001
Procalcitonin > 0.1 ng/ml	1.2 (1.0, 1.3) 0.033	1.1 (0.8, 1.5) 0.682	01.2 (1.0, 1.3) 0.021
C-reactive protein > 10 mg/liter	1.2 (1.1, 1.4) 0.003	1.2 (0.9, 1.8) 0.264	1.2 (0.9, 1.8) 0.264

Adjusted for age, Black, White, Asian, Latino, temperature, mean arterial pressure, oxygen saturation, MI, PVD, CVD, dementia, COPD, diabetes mellitus complicated and renal disease.

CHF, congestive heart failure; MI, myocardial infarction; PVD, peripheral vascular disease; CVD, cerebrovascular disease; COPD, chronic obstructive pulmonary disease; WBC, white blood cellcount; IL-6, interleukin-6.

## Discussion

We analyzed a large inpatient cohort with confirmed COVID-19 infection, evaluating the predictive value of systemic inflammatory indicators at admission for in-hospital mortality risk. As expected, a significant increase in inflammatory levels contributed to a higher in-hospital mortality risk, but this relationship existed in non-CHF patients rather than in CHF patients.

The COVID-19 pandemic has caused significant morbidity and mortality (Wang et al., 2020; Wu et al., 2023), which can also be complicated by acute or chronic cardiovascular syndromes, in addition to severe respiratory complications (Chung et al., 2021; Patone et al., 2022). These syndromes can manifest as acute coronary syndrome, myocarditis, cardiac arrhythmias, or clinical HF, with or without hemodynamic instability (Driggin et al., 2020; Sala et al., 2020; Heidecker et al., 2022; Zuin et al., 2023a). Such early or late cardiovascular complications may occur at any point during hospitalization or even after discharge, once respiratory symptoms have improved (Bhatraju et al., 2020; Fried et al., 2020).

Previous evidence has indicated that COVID-19 infection may result in adverse cardiovascular events attributable to a severe inflammatory cytokine storm (Guzik et al., 2020; Liu et al., 2020). This is consistent with our findings, which showed that high levels of systemic inflammation significantly increased the risk of in-hospital mortality.

In fact, a number of preexisting comorbidities or risk factors have been associated with worse clinical outcomes in patients with COVID-19 infection. For example, previous studies confirmed that older patients with COVID-19 tended to have a higher risk of requiring mechanical ventilation, composite death, and/or ICU admission compared with younger patients (Guan et al., 2020). Evidence from Chinese populations has shown that the presence of preexisting cardiovascular comorbidities can increase the disease severity of hospital-treated COVID-19 (Novel Coronavirus Pneumonia Emergency Response Epidemiology Team, 2020). These studies concluded that among patients with COVID-19, the fatality rate was much higher in those with comorbidities than in those without (Novel Coronavirus Pneumonia Emergency Response Epidemiology Team, 2020). Reported mortality rates included 10.5% for patients with cardiovascular disease (CVD), 6.0% for hypertension, 7.3% for diabetes, 6.0% for cancer, and 6.3% for chronic respiratory disease (Epidemiology Working Group for NCIP Epidemic Response, 2020; Novel Coronavirus Pneumonia Emergency Response Epidemiology Team, 2020; Ruan, 2020; The Novel Coronavirus Pneumonia Emergency Response Epidemiology Team, 2020). Clearly, the occurrence of CVD significantly increased the mortality rate to a greater extent than other preexisting comorbidities (Novel Coronavirus Pneumonia Emergency Response Epidemiology Team, 2020). These findings were also supported by a previous meta-analysis, which reported that CVD was present in 4.2% of the total population with COVID-19, and among these, 22.7% died (Epidemiology Working Group for NCIP Epidemic Response, 2020). However, there was large heterogeneity in reported death rates due to differences in study populations with various cardiovascular comorbidities. Another report from a Chinese population showed that HF was considered an adverse outcome in 23% of individuals with COVID-19; approximately 52% of non-survivors had HF, whereas only 12% of survivors had HF (Zhou et al., 2020). Similarly, in our study, CHF patients (27.73%) had a slightly higher in-hospital mortality rate than non-CHF patients (23.93%), but the statistical difference between the two groups was not significant (p = 0.053).

A previous meta-analysis involving patients who had recovered from COVID-19 (N = 21,463,173; mean age, 54.5 years; 58.7% male) was performed. This study concluded that the risk for incident HF may occur even in individuals at low cardiovascular risk, which was inconsistent with most previous conclusions (Zuin et al., 2023b). The authors explained that the relatively high heterogeneity might be due to differences in baseline population characteristics, preexisting CVD risk, vaccination status for COVID-19, or prior HF history. Our study also found that high systemic inflammatory levels at admission were significantly associated with an increased risk of in-hospital mortality in non-CHF patients but not in CHF patients. HF is well known as the terminal stage of heart disease. Many conditions—including diabetes, hypertension, coronary heart disease, valvular disease, and myocarditis—can lead to HF (Daugherty et al., 2021; Salah et al., 2022; Wang et al., 2022). The heterogeneity of these causes may partly explain the seemingly contradictory phenomena. Other possible sources of variation include differences in population selection by age and race, therapeutic drugs (β-blockers, ACE inhibitors, glucose-lowering, antihypertensive, and lipid-lowering agents), medical history, statistical methods, inclusion criteria, and other factors (Daugherty et al., 2021; Salah et al., 2022; Wang et al., 2022). For instance, previous studies, including meta-analyses, did not systematically analyze prior HF history, limiting the ability to further evaluate death risk (Daugherty et al., 2021; Salah et al., 2022; Wang et al., 2022). The pathological mechanism underlying the higher risk of death in CHF patients with COVID-19 has not yet been fully confirmed. Direct viral invasion causing myocardial damage or endothelial cell infection may partly explain the increased risk of worsening HF or death in most cases (Nishiga et al., 2020; Cowie et al., 2022). However, in our CHF patients, chronically elevated baseline inflammation was not significant, which may explain why inflammatory markers were not directly and significantly correlated with mortality in CHF patients in our analysis. More evidence from prospective cohort studies is needed to further validate our findings.

Undoubtedly, our study findings provide several implications for clinical practice. The risk of in-hospital mortality may be predicted by systemic inflammatory levels at admission during the acute phase of COVID-19 infection in non-CHF patients but not in CHF patients. Although controversies remain regarding mortality risk during hospitalization among CHF patients, our results support the conclusion that patients with preexisting CHF or a prior HF history may be at increased risk of in-hospital mortality. There is no doubt that the present study has several limitations. First, this was a retrospective study, and some variables—such as treatment during hospitalization (e.g., corticosteroids and antivirals), cardiac function, and cause of death—were missing, limiting further refinement of the study. Second, gender data were missing; given that previous studies have suggested that COVID-19 sequelae may differ by gender, this could introduce potential bias. Finally, although sensitivity analysis was performed in our study, additional subgroup analyses stratified by age, comorbidities, or biomarker levels should also be conducted to evaluate the robustness of these findings.

## Conclusion

Our findings showed that elevated inflammatory levels at admission in CHF patients were not associated with an increased risk of in-hospital mortality. However, higher inflammatory levels were associated with a greater risk of in-hospital mortality among non-CHF patients. More evidence is needed in the future to support these findings.

## Data Availability

The datasets presented in this study can be found in online repositories. Our study data were obtained from the Dryad database (https://doi.org/10.5061/dryad.7d7wm37sz).
